# A Double‐Blind Randomized Study of Two Doses of Oral Isotretinoin in the Treatment of Recalcitrant Facial Flat Warts: Impact on Quality of Life, Anxiety, and Depression

**DOI:** 10.1002/hsr2.70684

**Published:** 2025-04-21

**Authors:** María Guadalupe Olguín‐García, María Luisa Peralta‐Pedrero, Martha Alejandra Morales‐Sánchez, Elisa Vega‐Memije, Víctor Manuel Bautista‐de Lucio

**Affiliations:** ^1^ Programa de Maestría y Doctorado en Ciencias Médicas, Odontológicas y de la Salud Universidad Nacional Autónoma de México Mexico City Mexico; ^2^ Education and Research Department Centro Dermatológico Dr. Ladislao de la Pascua (CDP) Secretaría de Salud de la Ciudad de México Mexico City Mexico; ^3^ Internal Medicine Department Hospital General Dr. Darío Fernández Fierro, ISSSTE Mexico City Mexico; ^4^ Dermatology Department Hospital General Dr. Manuel Gea González Secretaria de Salud Mexico City Mexico; ^5^ Microbiology and Ocular Proteomics, Research Unit, Institute of Ophthalmology Fundación de Asistencia Privada Conde de Valenciana Mexico City Mexico

**Keywords:** oral isotretinoin, recalcitrant flat warts, treatment

## Abstract

**Background and Aims:**

Recalcitrant facial flat warts are caused by human papillomavirus and may persist for years despite treatment. Oral isotretinoin administered at a dose of 0.5 mg/kg/day is effective and safe. However, the adverse effects reported are dose‐dependent behaviors and they could limit their use. We aim to compare the efficacy and safety of two doses of oral isotretinoin for the treatment of recalcitrant facial flat warts and to assess the quality of life, anxiety, and depression in the individuals studied.

**Methods:**

Isotretinoin 0.3 mg/kg/day or isotretinoin 0.5 mg/kg/day was administered to 21 and 19 adult patients, respectively, in a double‐blind, randomized fashion for 12 weeks. Cutaneous lesions were assessed, and adverse events, including serologic changes, were recorded. It is considered that warts were recalcitrant if the patient was treated for at least 3 years with at least two of the following options: retinoids, 5‐fluorouracil, imiquimod, and cryotherapy. In addition, quality of life, anxiety, and depression were assessed at the beginning and end of follow‐up.

**Results:**

In the isotretinoin 0.3 mg/kg/day group, 35% of the patients had a complete response, and 66% had a partial response, while in the isotretinoin 0.5 mg/kg/day group, 73.7% presented a complete response, and 26.31% presented a partial response (*p* = 0.015). The most frequent adverse event was cheilitis. There was an elevation of aspartate aminotransferase (*p* = 0.020) and total bilirubin (*p* = 0.015) in the isotretinoin 0.5 mg/kg/day group. Improvement in the quality‐of‐life score (*p* = 0.0001) and a reduction in the anxiety (*p* = 0.00) score was observed in both groups.

**Conclusion:**

Oral isotretinoin at a dose of 0.5 mg/kg/day is safe and effective for the treatment of recalcitrant facial flat warts in adults, with lower recurrence rates than 0.3 mg/kg/day. Prolonged treatment with isotretinoin for over 12 weeks in adults can increase the overall response rate.

**Trial Registration:** Registry of ClinicalTrials.gov identifier: NTC04290572; https://classic.clinicaltrials.gov/.

## 1 Introduction

Facial flat warts are small infectious neoformations of the skin caused by Subtypes 3, 10, 27, 28, 38, and 49 of the human papillomavirus (HPV) [1, 2]. Although two‐thirds of cases may present spontaneous involution after 2 years [3], up to 30% may become recalcitrant [4], which makes the treatment of recalcitrant facial flat warts a challenge for dermatologists [5, 6].

Oral isotretinoin has been used successfully for the treatment of different types of warts at different doses [7, 8, 9]; without an established dosing regimen to date [10, 11]. It is known that oral isotretinoin administered at a dose of 0.5 mg/kg/day for 12 weeks is effective and safe for the treatment of recalcitrant facial flat warts [11]. However, oral isotretinoin has different adverse effects reported [12, 13, 14, 15, 16, 17].

All adverse effects have a dose‐dependent behavior [11]. For this reason, some authors have reduced the dose of isotretinoin without affecting efficacy [18, 19] while others [10] disagree about the 100% efficacy of oral isotretinoin at low doses.

The present study compares the efficacy and safety of two doses of oral isotretinoin for the treatment of recalcitrant facial flat warts in adult patients, in whom their quality of life, anxiety, and depression are assessed.

## 2 Materials and Methods

### 2.1 Study Design

The current study is a randomized, double‐blinded, and isotretinoin 0.3 mg/kg/day or 0.5 mg/kg/day‐controlled study performed in Mexico City at the Dermatologic Center “Dr. Ladislao de la Pascua.” Clinical trial conducted between June 2022 and July 2023. In total, 40 adults were enrolled in the study.

Participants were randomly assigned to receive either isotretinoin 0.3 mg/kg/day or 0.5 mg/kg/day. Digital photographs were taken, and clinical evaluation was performed on Days 1 (baseline visit), 28, 56, and 84. Additionally, laboratory assessments, including a complete blood cell count, liver function tests, lipid profile, and creatine phosphokinase (CPK) levels, were carried out on Days 1, 28, and 84. This rigorous monitoring process ensured the collection of accurate and reliable data.

Quality of life, anxiety, and depression were also assessed. Therefore, before and at the end of the intervention, patients answered the Spanish version of the Dermatology Life Quality Index (DLQI) [20], which was developed to assess people's quality of life. It contained 10 items about the participant's recent feelings and aspects of skin disorder. It addressed symptoms (itching, pain, and irritation), emotions (embarrassment, distress, and anger), daily activities (shopping and housework), clothing, social or leisure activities, physical activity, educational opportunities, sexual behavior, personal relationships (with partner, friends, and relatives), and treatment options.

The Beck Anxiety Inventory (BAI) [21], which is a valuable tool for assessing somatic symptoms of anxiety in both anxiety disorders and depressive conditions, was applied. The questionnaire consisted of 21 questions, providing a range of scores between 0 and 63. The Beck Depression Inventory (BDI) [22] was also executed, which consisted of 21 multiple‐choice questions with a minimum score of 0 and a maximum of 63. The higher the score, the greater the probability of depression; a score of 1–10 is considered normal. These instruments are used first to screen for psychiatric comorbidities and then to assess the effect of interventions on these dimensions.

### 2.2 Inclusion and Exclusion Criteria

The inclusion criteria for the study were: being more than 18 years of age and possessing a histologically confirmed diagnosis of facial flat warts, as well as a diagnosis of warts for at least 3 years. In addition, having used at least one of the following treatments without complete clearance of the lesions: tretinoin, imiquimod, 5‐fluorouracil, salicylic acid, cryotherapy with liquid nitrogen, cimetidine, and zinc sulfate, was also valued. Topical treatment had to be withheld for at least 3 weeks before the start of the study.

Only patients with average values in liver function tests and lipid profiles were included. Female patients were recruited only if they were not pregnant and agreed to use a contraception method during the trial, either hormonal or an intrauterine device. Patients with Sjögren syndrome and renal and hepatic diseases were excluded. The criteria for discontinuation of therapy were pregnancy and an elevation in transaminases, cholesterol, and triglycerides two or more times higher compared to baseline and CPK three or more times higher compared to baseline.

### 2.3 Randomization and Blinding

Patients were randomly assigned to the groups using a sequence generated by the program https://www.sealedenvelope.com/. To ensure allocation concealment, a person not engaged in the study conducted the blocking and allocation sequence. Each group was assigned a unique code, which was only known to the research assistant. The researchers were blinded regarding the medicine.

### 2.4 Intervention

**Figure 1:**
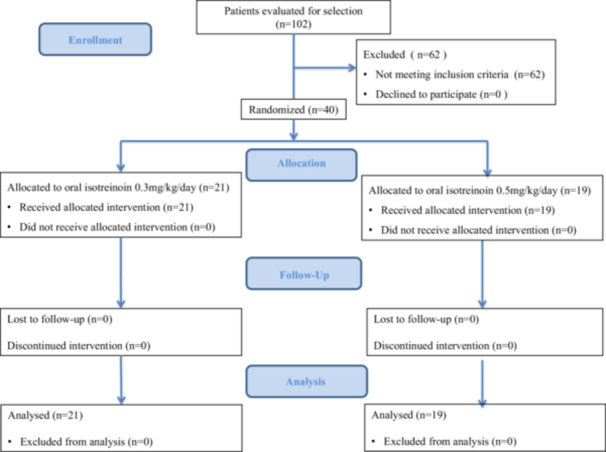
Diagram flow of patients in the study.

Patients in Group 1 received isotretinoin 0.3 mg/kg/day, and those in Group 2 received 0.5 mg/kg/day, both for 12 weeks (Figure 1). All patients signed the written informed consent.

The intervention was suspended if there was an elevation of transaminase, cholesterol, or triglyceride values 2 levels above the baseline, elevation of CPK values 3 levels above the baseline, or withdrawal of contraceptive method or pregnancy.

### 2.5 Primary and Secondary Goals

The primary goal of the study was to evaluate the reduction in the number of warts at Week 12. A secondary endpoint was the percentage of patients with complete removal of warts at the end of the study. A dermatologist who was blinded to treatment group assignments performed efficacy assessments, wart counts, and digital photography. A complete response was considered when 100% of the warts disappeared. Safety endpoints included the incidence of adverse events and laboratory evaluations throughout the study.

In addition, quality of life, anxiety, and depression were assessed at the beginning and end of follow‐up.

### 2.6 Sample Size and Statistical Analysis

The sample size was calculated using the program G*Power 3.1.9.4 to compare proportions. For complete remission, there would be a difference of at least 40% between groups: 29 patients/group plus 20% losses, so 34 patients/group would be included. Data were analyzed using the SPSS v.25 program.

The baseline characteristics were described with proportions, medians, and percentiles. For differences between study groups in terms of the number of lesions and laboratory values, we used the Mann–Whitney *U* test and the Student's *t*‐test. Wilcoxon test was used to evaluate the difference in the number of warts before and after the interventions, and Fisher's exact test was used to compare the proportion of patients with complete responses between the groups. Student's *t*‐test was used to determine if there was a difference in the reduction of warts between groups. Results were considered to be statistically significant if the *p* value was < 0.05.

## 3 Results

The study was completed on 21 patients (16 females, 5 males; median age 29 years) in the isotretinoin 0.3 mg/kg/day group and 19 patients (12 females, 7 males; median age 28 years) in the isotretinoin 0.5 mg/kg/day group.

Only 10% of patients in both groups had received the HPV vaccine. The median duration of warts was 4 years in Group 1 treatment, interquartile range (IQR) 3.5 years, and the median duration of warts was 5 years in Group 2 treatment, IQR 3.10 years (*p* = 0.146).

At least 42.9% of patients in Group 1 had one and two previous treatments, and 14.3% of patients had three previous treatments compared with 52.6% of patients in Group 2 had one, 26.3% had two, and 21.1% had four previous treatments (*p* = 0.538) (Table 1). The most common previous treatment in both groups was tretinoin, followed by cryotherapy, salicylic acid, and 5‐fluorouracil, without significant differences between the two treatment groups (*p* > 0.348).

**Table 1:** Baseline characteristics of treatment groups.

	Isotretinoin 0.3 mg/kg/day *n* = 21 (%)	Isotretinoin 0.5 mg/kg/day *n* = 19 (%)	*p* b
Sex			
Female	16 (76.2)	12 (63.2)	0.369
Male	5 (23.8)	7 (36.8)	
Age, years^a^	29.57 (8.77)	28.95 (6.64)	0.803^c^
HPV vaccine			
Yes	2 (9.5)	2 (10.5)	0.916
No	19 (90.5)	17 (89.5)	
Warts evolution time^a^ (years)	4 (3, 5.5)	5 (3, 10)	0.146^c^
Previous treatments			
1	9 (42.9)	10 (52.6)	0.538
2	9 (42.9)	5 (26.3)	
3	3 (14.3)	4 (21.1)	

Abbreviation: HPV = human papillomavirus.

a Median and interquartile range.

b *χ* ^2^ test.

c Mann–Whitney *U* test.

**Figure 2:**
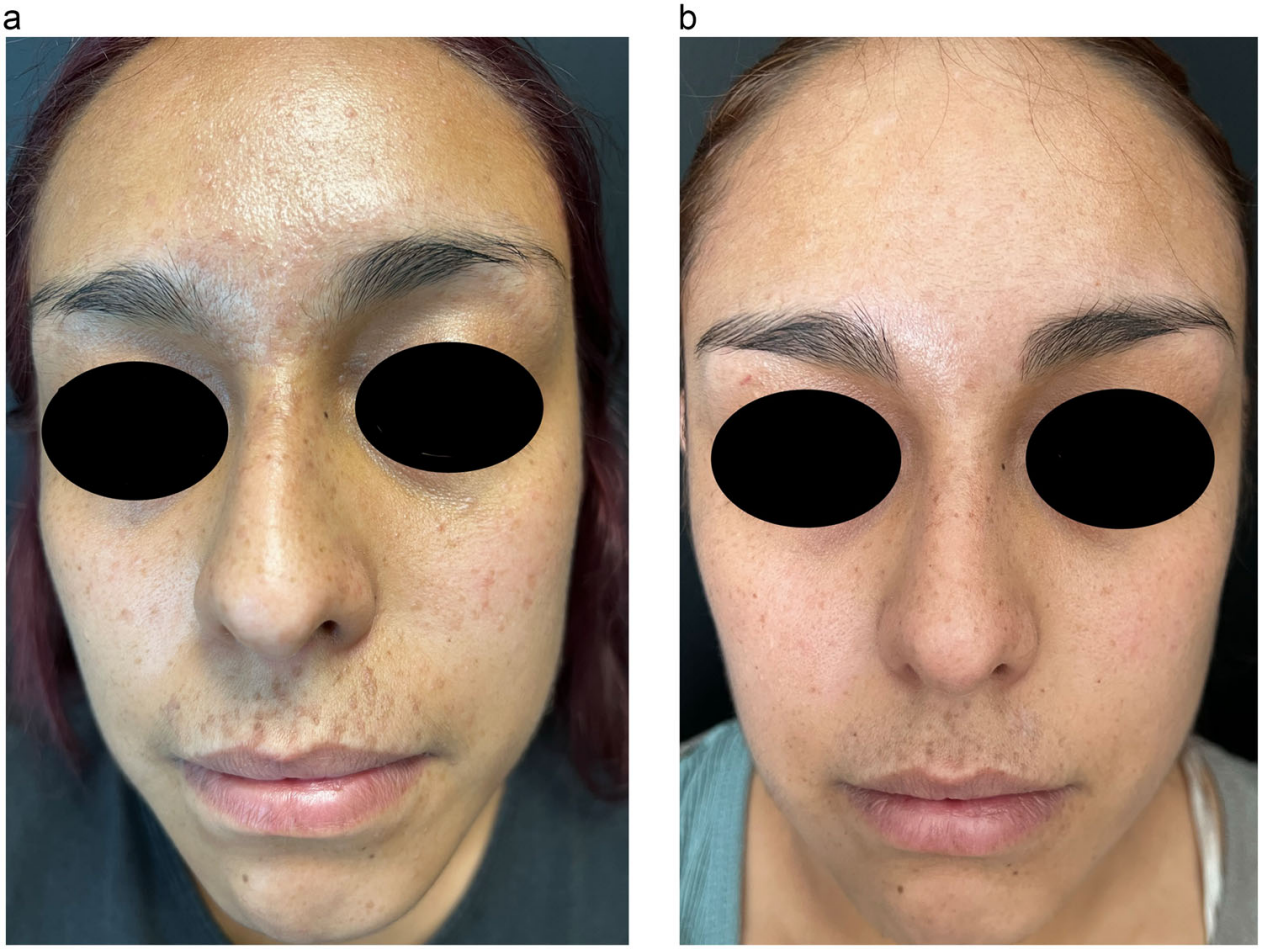
(a) A 24‐year‐old female with 48 recalcitrant facial flat warts during 4 years with topical retinoid, 5‐fluorouracil, and cryosurgery as previous treatments. (b) After treatment with oral isotretinoin at 0.3 mg/kg/day for 12 weeks.

**Figure 3:**
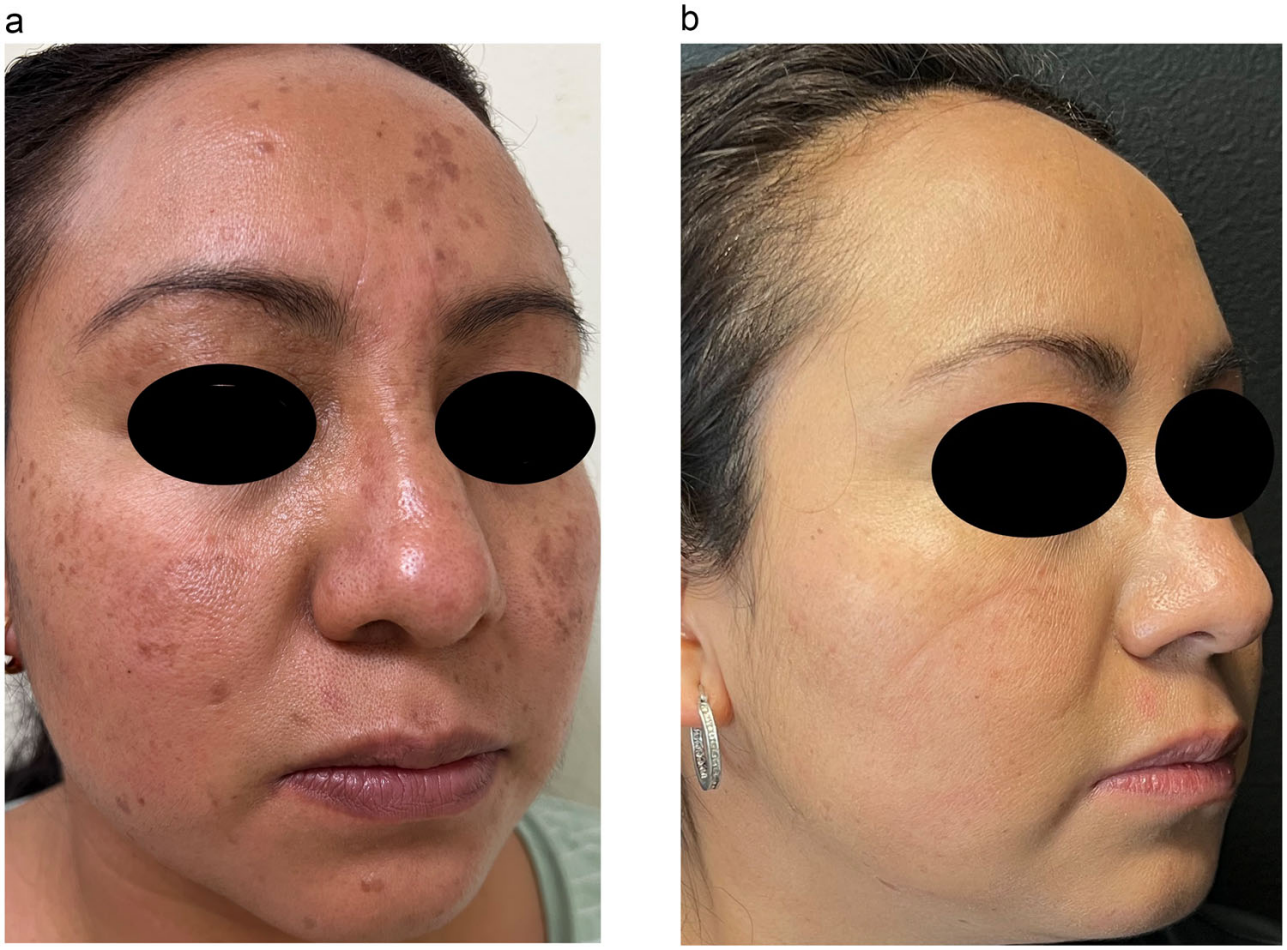
(a) A 29‐year‐old female with 51 recalcitrant facial flat warts during 5 years with topical retinoid, imiquimod, and cryosurgery as previous treatments. (b) After treatment with oral isotretinoin at 0.5 mg/kg/day for 12 weeks.

At the end of the follow‐up, the median reduction in the number of warts was 28 in Group 1 and 50 in Group 2, those who received a higher dose of isotretinoin had fewer warts compared to their baseline (*p* = 0.024) (Figures 2a,b and 3a,b). A significant difference was observed in the percentage of patients who did not have lesions after 12 weeks of treatment: in the isotretinoin 0.3 mg/kg/day group, 7 (35%) had a complete response and 14 (66%) had a partial response out of 21 patients included, while in the isotretinoin 0.5 mg/kg/day group, 14 (73.7%) presented a complete response and 5 (26.31%) presented a partial response of the 19 patients included (*p* = 0.015) (Table 2). The Relative Risk (RR) of the 0.5 mg/kg/day dose compared to the 0.3 mg/kg/day dose was 2.1053 95% CI 1.0936–4.0526.

**Table 2:** Effect of the intervention on the number of warts.

	Isotretinoin 0.3 mg/kg/day *n* = 21 Md (IQR)	Isotretinoin 0.3 mg/kg/day *n* = 19 Md (IQR)	*p*
Number of warts baseline	29 (20–64.5)	50 (37–92)	
	Min 15 Max 163	Min 21 Max 198	0.03
Number of warts at the end	2.5 (0–4) Min 0 Max 22	0 (0.3) Min 0 Max 100	0.066
Warts at the end			
Yes	13 (65)	5 (26.3)	0.015^a^
No	7 (35)	14 (73.3)	
Difference of warts	−28 (−65, −19)	−50 (−92, −37)	0.024

*Note:* Mann–Whitney *U* test.

Abbreviations: IQR = interquartile range; Min = minimum; Max = maximum; Md = median or Q2.

^a^ *χ* ^2^ test.

Table 3 shows the clinical side effects presented during the study; there were no significant differences between groups and none of the patients required discontinuation of the treatment. Cheilitis was present in 95% of cases in both groups during the 12 weeks of treatment, followed by dry skin (77%), pruritus (59%), and dry eyes (56%). Subjects reported 100% adherence to treatment, confirmed by pill counts on each visit.

**Table 3:** Side effects in treatment groups.

	Isotretinoin			Isotretinoin		
	0.3 mg/kg/day % (*n* = 21)			0.5 mg/kg/day % (*n* = 19)		
	4 weeks	8 weeks	12 weeks	4 weeks	8 weeks	12 weeks
Cheilitis	95.2 (20)	95.2 (20)	95.2 (20)	100 (19)	94.7 (18)	89.5 (17)
Pruritus	61.9 (13)	47.6 (10)	47.6 (10)	63.2 (13)	36.8 (7)	31.6 (6)
Dry skin	71.4 (15)	61.9 (13)	71.4 (15)	78.9 (15)	68.4 (13)	31.6 (6)
Dry eyes	52.4 (11)	57.1 (12)	52.4 (11)	52.6 (10)	31.6 (6)	31.6 (6)
Rash	52.4 (11)	28.6 (6)	19 (4)	26.7 (5)	21.1 (4)	5.3 (1)
Muscle pain	52.4 (11)	23.8 (5)	42.9 (9)	47.4 (9)	36.8 (7)	26.3 (5)
Headache	42.9 (9)	28.6 (6)	33.3 (7)	26.3 (5)	31.6 (6)	36.8 (7)
Diffuse alopecia	33.3 (7)	33.3 (7)	38.1 (8)	10.5 (2)	21.1 (4)	15.8 (3)
Photosensitivity	33.3 (7)	28.6 (6)	42.9 (7)	52.6 (10)	36.8 (7)	26.3 (5)
Epistaxis	23.8 (5)	33.3 (7)	33.3 (7)	47.4 (9)	26.3 (5)	31.6 (6)
Impaired vision^a^	28.6 (6)	33.3 (7)	42.9 (7)	26.3 (5)	26.3 (5)	15.8 (3)

a Visual acuity.

There were no significant differences between groups in the baseline laboratory investigations. But there was an elevation of the following laboratory studies in the isotretinoin 0.5 mg/kg/day group: aspartate aminotransferase (AST) at the 4 (*p* = 0.020) and 12 (*p* = 0.035) weeks of treatment and the total bilirubin (TB) (*p* = 0.015) when we compared baseline levels with those at the end of the study.

Table 4 shows the scores on the DLQI, BAI, and BDI presented during the study. There were no significant differences between groups in the baseline and at the end of the follow‐up. However, a significant improvement in DLQI score was observed in both groups (*p* = 0.0001), as well as a reduction in BAI score (*p* = 0.00) for the 0.3 mg/kg/day group and (*p* = 0.022) for the 0.5 mg/kg/day group. Concerning the BDI score, a significant difference was observed in the 0.3 mg/kg/day group (*p* = 0.020) but not in the 0.5 mg/kg/day group (*p* = 0.138).

**Table 4:** Quality of life, anxiety, and depression scores in the treatment groups.

	Isotretinoin 0.5 mg/kg/day Md (p25–p75) *n* = 21	Isotretinoin 0.5 mg/kg/day Md (p25–p75) *n* = 19	*p* a
1. DLQI	8 (6–10.5)	10 (5–5)	0.592
2. DLQI	0.5 (0–1)	0 (0–3)	0.708
*p* b	0.0001	0.0001	
1. BAI	9 (1.5–14)	5 (0–9)	0.117
2. BAI	3.5 (0–6.75)	0 (0–7)	0.283
*p* b	0.001	0.022	
1. BDI	3 (0.5–8)	0 (0–3)	0.078
2. BDI	0.5 (0–4.0)	0 (0–2)	0.194
*p* b	0.020	0.138	

Abbreviations: BAI = Beck Anxiety Inventory; BDI = Beck Depression Inventory; DLQI = Dermatology Life Quality Index; Md = median; p25 = percentile 25; p75 = percentile 75.

^a^ Mann–Whitney *U* test.

^b^ Wilcoxon test.

Recurrence of lesions was observed in 3 (42.82%) of the 7 patients with complete response in the 0.3 mg/kg/day dose group and in 1 (7.14%) of the 14 patients with complete response in the 0.5 mg/kg/day oral isotretinoin group at 4 months follow‐up.

## 4 Discussion

In this study, we compared two doses of oral isotretinoin for the treatment of recalcitrant facial flat warts; the results confirm that with the dose of 0.5 mg/kg/day, the percentage of complete remission of flat warts is higher than with the dose of 0.3 mg/kg/day (73.7% vs. 35%). This benefit is seen in the RR obtained by patients with the higher dose of isotretinoin, who had more than twice the complete remission of flat warts than patients receiving 0.3 mg/kg/day.

This complete response to the higher dose of isotretinoin is similar to that described by Al‐Hammamy et al. [13] (73.07%), Kaur et al. [14] (69%), and Nofal et al. [23] (76%), who used a higher dose (0.6 mg/kg/day). Although complete remission in these studies is similar, there are some differences with our study. Al‐Hammamy et al. [13] indicate treatment for only 8 weeks, and Kaur et al. [14] and Nofal et al. [23] indicate treatment for 3 months or until a complete response is achieved, whichever comes first. In our study, we only included adult patients with warts who had at least 3 years of evolution. Al‐Hammamy et al. [13] and Nofal et al. [23] include patients from 5 to 6 years of age, respectively; furthermore, although Nofal et al. [23] include patients with flat warts (21/100 patients) and comment that it was the second best‐responding group, he does not specify the point percentage of complete response to the dose he calls high dose of oral isotretinoin; so, 76% is the full response of all patients: vulgar, plantar, plantar and genital. Another difference is that these studies included some patients who had < 2 years of evolution with flat warts. Our decision to include patients with warts that have evolved for more than 3 years was to reduce the possibility of spontaneous regression of the lesions, which has been documented in some patients during the first 2 years of disease [24, 25].

Concerning the percentage of complete response obtained by our patients with the dose of 0.3 mg/kg/day (35%), it is lower than that reported by Nofal et al. [26] (44.4%) in his study published in 2020, where he included 108 patients with flat warts; of which 36 were assigned to receive only oral isotretinoin at this lower dose and of (46%) in the author's most recent study [23], where he includes 11 patients with flat warts to receive this dose. The difference with our study may lie in the first case, in the more significant number of patients included the time of evolution with lesions (1–36 months), and the age range of the patients included (4–44 years). In the second case, the time of evolution with lesions (1–5 years), the age range of the patients included (5–45 years), and the actual percentage of complete response to this dose in the 11 patients studied may have an influence. Regardless of all the differences discussed, what is clear is that the highest percentage of complete response is obtained with higher doses of isotretinoin, 0.5 mg/kg/day in our study and 0.6 mg/kg/day in Nofal's study.

When analyzing the risk/benefit ratio of both interventions, we observed no significant differences between the groups in terms of the occurrence of adverse events; similarly, Nofal et al. [23] found no significant differences between the two intervention groups. We agree that cheilitis is an adverse event that occurs in all patients, in addition to dry skin, dry eyes, pruritus, headache, and muscle pain [11, 13, 14, 15, 26]. Despite the presence of adverse effects in both groups, none of the patients dropped out of the study, and all completed the 12 weeks of treatment, unlike Kaur et al. [14], who described four losses due to severe cheilitis and polymenorrhea. Nofal et al. [23] have no losses but also describe menstrual alterations as adverse events. None of the women studied presented these alterations, as reported by Chelliah and Glass [27] in their 2020 review; however, it would be interesting to evaluate these results in more extensive prospective studies. It is essential to remember that women with childbearing potential should be prescribed a method of contraception during the whole treatment with isotretinoin [28]. In agreement with that reported by Pona et al. [16], in this study, we observed elevated transaminases and bilirubin, with a significant difference in both treatment groups in favor of the higher dose.

In 2023 [11], we presented a patient whose dose of oral isotretinoin was reduced to 15 mg/day due to increased levels of ALT, AST, CPK, cholesterol, and triglycerides, with complete remission of the lesions after 12 weeks of treatment, no recurrence. However, in the present study, unlike other authors who have used low doses of isotretinoin [18, 19], we observed a recurrence of lesions in 42.86% of patients. This recurrence is even higher than that described by Nofal et al. [23], for a dose of 0.3 mg/kg/day and similar to ours when using a higher dose (7.8%). Notably, in studies where 0.5 mg/kg/day of oral isotretinoin was indicated, recurrence was even higher (21% and 36%). Perhaps because the age range is more significant in these studies, and even pediatric patients were administered.

Bremner et al. [29] describe an association between the use of oral isotretinoin and psychiatric side effects; other authors comment that the therapeutic dose does not increase these risks and that they even improve when the underlying pathology improves [30], a comment with which we agree because although in our study there are no significant differences between DLQI, BAI, and BDI scores when comparing both intervention groups, there is statistical significance in each group before and at the end of treatment. More than 80% of the patients in both groups improved their quality of life and decreased their anxiety and depression indexes. Similar results are presented by Marron et al. [31] with significant improvements in anxiety (*p* < 0.001), depression (*p* < 0.005), and quality of life scales.

As far as we know, this is the first randomized clinical trial for treating recalcitrant facial flat warts with oral isotretinoin that addresses these three dimensions. Although the treatment time, in this case, is short, we agree that patients should be closely monitored to identify those at high risk of developing psychiatric symptoms [32].

It is important that all patients receiving oral isotretinoin at a dose of 0.5 mg/kg/day be prescribed the application of emollients like petroleum gels, petroleum jelly, or saline on the nares, use of ocular lubricants, sunscreen, and regular checkups and blood test [33].

In this study, a sample size was calculated to recruit 35 patients/group. However, the interim analysis showed a clinically and statistically significant difference in favor of the group who took the 0.5 mg/kg/day dose, so we decided to report these results.

One significant limitation among all the others in this study is that patients were only followed for 4 months after the clinical trial was concluded.

## 5 Conclusion

Oral isotretinoin at a dose of 0.5 mg/kg/day is safe and effective for treating recalcitrant facial flat warts in adults, with lower recurrence rates than 0.3 mg/kg/day. Prolonged treatment with isotretinoin for over 12 weeks in adults can increase the complete response rate. There is no evidence to support the use of isotretinoin in pediatric patients because controlled clinical trials are needed to demonstrate that its efficacy is superior to its adverse effects.

## Author Contributions

**María Guadalupe Olguín‐García:** conceptualization, data curation, formal analysis, investigation, methodology, project administration, resources, validation, writing – original draft, writing – review and editing. **María Luisa Peralta‐Pedrero:** formal analysis, investigation, methodology, project administration, supervision, validation, writing – review and editing. **Martha Alejandra Morales‐Sánchez:** conceptualization, formal analysis, methodology, supervision, validation, writing – review and editing. **Elisa Vega‐Memije:** resources, supervision, validation, visualization. **Víctor Manuel Bautista‐de Lucio:** investigation, validation, visualization, resources.

## Ethics Statement

The trial was conducted according to the ethical guidelines of the Declaration of Helsinki and performed according to the Good Clinical Practice guidelines. The Ethical and Research Committee of the center approved the study code 176/2019 and the Ethical Committee of the Secretaria de Salud de la Ciudad de Mexico CONBIOETICA Code: 101‐010‐007‐20. Registry of ClinicalTrials.gov identifier: NTC04290572. https://classic.clinicaltrials.gov/.

## Consent

Informed consent has been obtained from the patient.

## Conflicts of Interest

The authors declare no conflicts of interest.

## Transparency Statement

The lead author María Luisa Peralta‐Pedrero affirms that this manuscript is an honest, accurate, and transparent account of the study being reported; that no important aspects of the study have been omitted; and that any discrepancies from the study as planned (and, if relevant, registered) have been explained.

## Data Availability

The data that support the findings of this study are available from the corresponding author upon reasonable request.
